# Selective Atomic-Level Etching on Short S-Glass Fibres to Control Interfacial Properties for Restorative Dental Composites

**DOI:** 10.1038/s41598-019-40524-7

**Published:** 2019-03-07

**Authors:** Kiho Cho, Guannan Wang, Raju, Jian Fang, Ginu Rajan, Martina H. Stenzel, Paul Farrar, B. Gangadhara Prusty

**Affiliations:** 1 School of Mechanical and Manufacturing Engineering, University of New South Wales, Sydney, NSW 2052 Australia; 2 School of Chemistry, University of New South Wales, Sydney, NSW 2052 Australia; 3 Institute for Frontier Materials, Deakin University, Geelong, VIC 3220 Australia; 4 School of Electrical, Computer and Telecommunications Engineering, University of Wollongong, Wollongong, NSW 2522 Australia; 5 SDI Limited, VIC 3153 Bayswater, Australia

## Abstract

Interfacial bonding between fibre and matrix is most critical to obtain enhanced mechanical properties of the resulting composites. Here we present a new surface tailoring method of selective wet etching and organosilicon monomers (3-(Trimethoxysilyl) propyl methacrylate, TMSPMA) deposition process on the short S-Glass fibre as a reinforcing material, resulting in increased mechanical retention and strong chemical bonding between glass fibres and polymer resin (a mixture of triethylene glycol dimethacrylate (TEGDMA) and urethane dimethacrylate (UDMA) monomers). The effect of surface modification on fibre matrix interfacial strength was investigated through microdroplet tests. An S-Glass fibre treated with piranha solution (a mixture of H_2_O_2_ and H_2_SO_4_) for 24 hours followed by TMSPMA surface silanization shows highest increase up to 39.6% in interfacial shear strength (IFSS), and critical fibre length could be reduced from 916.0 µm to 432.5 µm. We find the optimal surface treatment condition in that the flexural strength of dental composites reinforced by the S-Glass fibres enhanced up to 22.3% compared to the composites without fibre surface treatments. The significant elevation in strength is attributed to changes in the surface roughness of glass fibres at atomic scale, specifically by providing the multiplied spots of the chemical bridge and nano-mechanical interlocking. The findings offer a new strategy for advanced tailoring of short S-Glass fibres to maximise the mechanical properties of biomedical and dental composites.

## Introduction

The unique functionality and enhanced physical and mechanical properties of the fibre reinforced composites (FRCs) are determined primarily by the properties of reinforcement fillers. Depending on different fibre types, fibre orientation and architecture, and dimensional size of a filament, polymer-based FRCs can have a wide range of properties in the applied systems^1–4^. Glass fibres have been among the topmost reinforcement fillers that are impregnated with a monomer or polymer matrix. Their high mechanical strength, electrical/chemical resistance, and dimensional stability in various harsh environments, including biological systems, make them extensively adapted in a growing number of applications, such as automotive, naval, aeronautical, and biomedical components. Regardless of fibre size in the fibre reinforced composites, surface morphology at the micro-and nanoscale and chemical reactivity along the fibre-matrix interface have a significant effect on bulk properties of composites^5–8^. Accordingly, a strong interfacial bonding between fibre and matrix is considered as an essential property of FRCs to effectively transfer the applied load to the fibres through the fibre-matrix interface and thereby experiencing similar stress on the fibre and the matrix^9,10^. As a functional interlayer, silane coupling agent (SCA) coating improves compatibility between organic and inorganic materials resulting an improvement in strength and toughness^11,12^. Incorporation of silica nanoparticles in the coating (size) on the glass fibres modified the fibre surface texture at the microscale and yielded significantly increased strength and energy absorption level in a fibre-epoxy composite system^8^. Recently the effect of intermediate coating thickness on the interfacial shear strength (IFSS) has been reported by using electrophoretic carbon nanotubes (CNTs) deposition method on the S-Glass fibre^7,13^. The interfacial properties can be tailored by controlling the surface morphology and thickness of the intermediate layer at nano- to microscale.

Glass fibres are basically made from silica-based glass and several metal oxides. The metal oxides are mixed to silica that melts to offer excellent thermal, electrical and chemical resistance and high mechanical strength. S-Glass fibres offer higher mechanical strength compared to other types of glass fibres and contains 64–66% of SiO_2_, 24–25% of Al_2_O_3_ and 9.5–10% of MgO^14^. S-Glass is studied for medical structural materials in orthopaedic implantation and dental restoration^15–17^ as they provide sufficient strength that is comparable to bone and tooth. Due to the long-lasting chemical stability and non-cytotoxicity^18^, glass fibres constitute the essential part of composite biomaterials, specifically for hard-tissue applications. The composite of polyetheretherketone (PEEK) and glass fibres provided a favourable surface environment for the proliferation of human bone cell indicating good biocompatibility *in vitro*^19^.

In biomedical and dental applications, long continuous fibres have a significant disadvantage in designing complex geometry structure and to fabricate small sized components due to the restriction of forming methods. They have somewhat limited application to simple components with anisotropic characteristics along the fibre direction. Hence, micro/nano-particle fillers have been most obvious candidates to develop the multi-functional and quasi-isotropic composite biomaterials^20–22^. Spherical or cluster shaped fillers have less geometric surface area compared to the short cylindrical fibres, and as the mechanical properties of composites are linked to interfacial contact area, cylindrical fibres have potential advantages to obtain a high mechanical performance of composites. To date, limited research have been attempted to utilise discontinuous glass fibres, with aspect ratios (ARs, a ratio of length to diameter) range from 5 to 640, in dental polymeric composites^17,23–25^. Moreover, for light curable polymer composites in dental applications, glass fibres and resin possess closer refractive indices that increase the light curing effectiveness and polymerisation rate. It also makes the glass fibre composite aesthetic unlike other fillers such as yellow coloured aramid fibre and black coloured carbon fibres or nanotubes^26,27^. The development of new restorative dental composites can have huge potential for future applications.

In this research, we developed a new mechanical and chemical surface tailoring method based on the atomic-level selective metal etching and organosilicon monomer grafting process on the uniformly chopped short S-Glass fibres. The effect of surface treatments on the interfacial properties of S-Glass fibre and dental resin were investigated using microdroplet pull-out tests and single fibre tensile tests. Highly promoted mechanical properties of fibre reinforced composites are measured by the 3-point bend tests and the reinforcement mechanisms are verified through the microscopic investigation of the fracture surface of composites. We purposely employed dental resin monomers as the base matrix in the composite system as it is biologically safe, light curable and has improved functionalities.

## Results

### Selective etching of metal oxides on the S-Glass fibres

**Figure 1:**
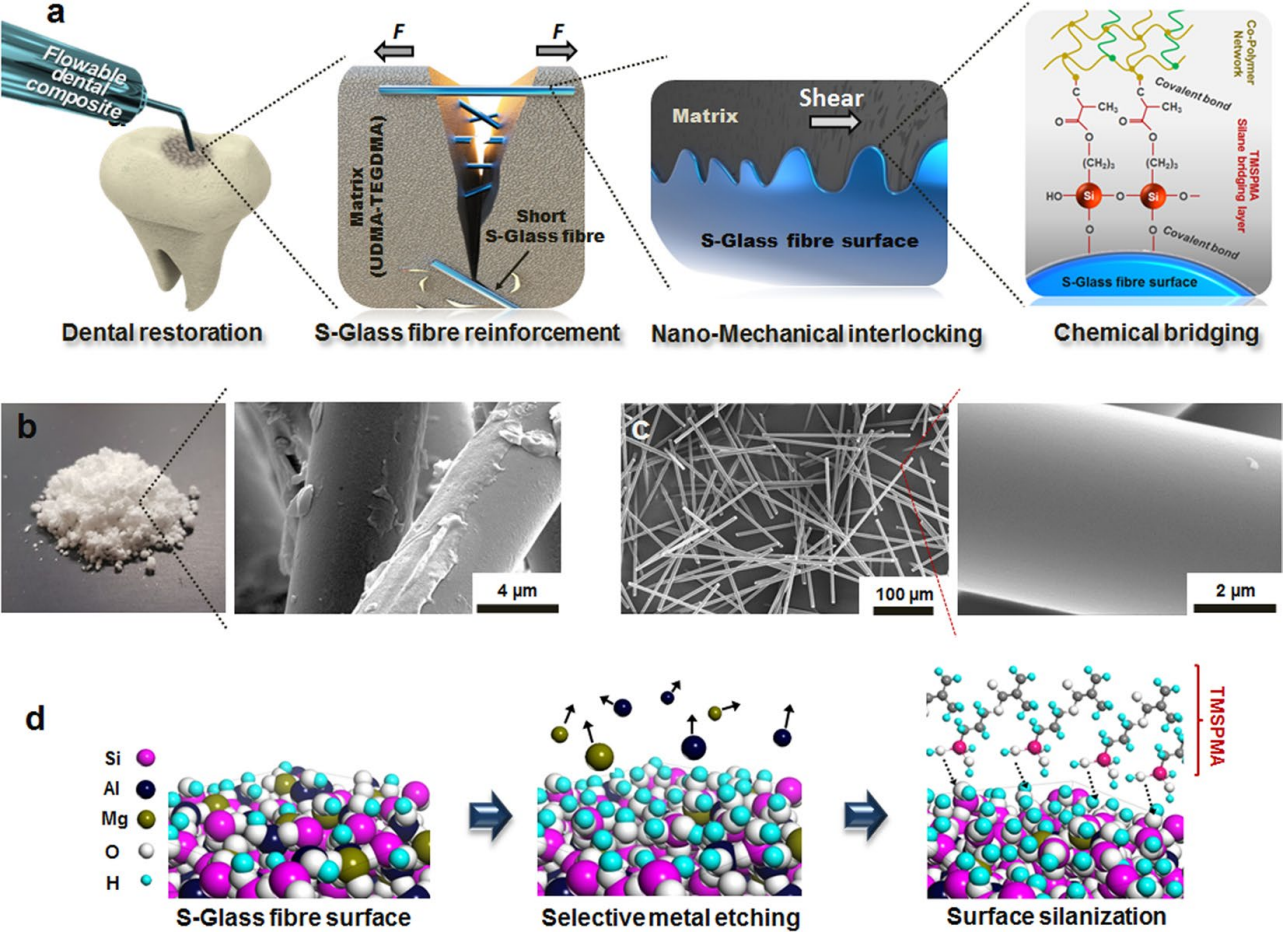
A design strategy for strong interfacial bonding of polymer resin to S-Glass fibre. (**a**) Schematic illustration showing multiscale structural evolution of short S-Glass fibre reinforced composite in a molar tooth. Randomly oriented glass fibres reinforce the composite with fibre bridging and crack deflection. The covalent bonds of the crosslinked polymer matrix are formed on the glass fibre through the SCA by chemically anchoring the organic polymer matrix to the inorganic glass fibre. (**b**) As-received short S-Glass fibres and SEM image of the short S-Glass fibres before the surface functionalisation process and (**c**) The SEM images of the short S-Glass fibres after the surface etching process. (**d**) The metal elements on the near surface of S-Glass fibre is selectively etched with acid solutions increasing the surface area and creating more hydroxyl groups (-OH) on the fibre. The entirely hydroxylated surface allows forming a uniform coating of SCAs which maintains cohesion of organic-inorganic interface.

The multiscale structure of the dental composites reinforced by surface functionalised S-Glass fibres is shown in Fig. 1a. As received glass fibres have a thick coating with surface modifiers or ‘size’ to protect the fibre from the static friction damage during manufacturing (Fig. 1b and see Supplementary Fig. 1a,b). The size coating was removed by etching in 37% HCl solution for 30 minutes in magnetic stirring until it completely dissolved (Supplementary Fig. 1c,d), and small debris and particles were filtered through centrifugation at 6,000 rpm. Then, the fibre average length and diameter were measured under field emission-scanning electron microscopy (FE-SEM). Statistical value of length and diameter exhibits uniform distribution with a mean value of 246.9 ± 19.5 *μ*m and 5.18 ± 0.26 *μ*m, respectively (see Supplementary Fig. 1e,f), and an AR of 48 ± 6. Chemical components of S-Glass fibres were analysed by SEM energy-dispersive spectrometry (EDS). As we found, they consist of SiO_2_ (65%), Al_2_O_3_ (25%) and MgO (10%) (see Supplementary Fig. 2).

**Figure 2:**
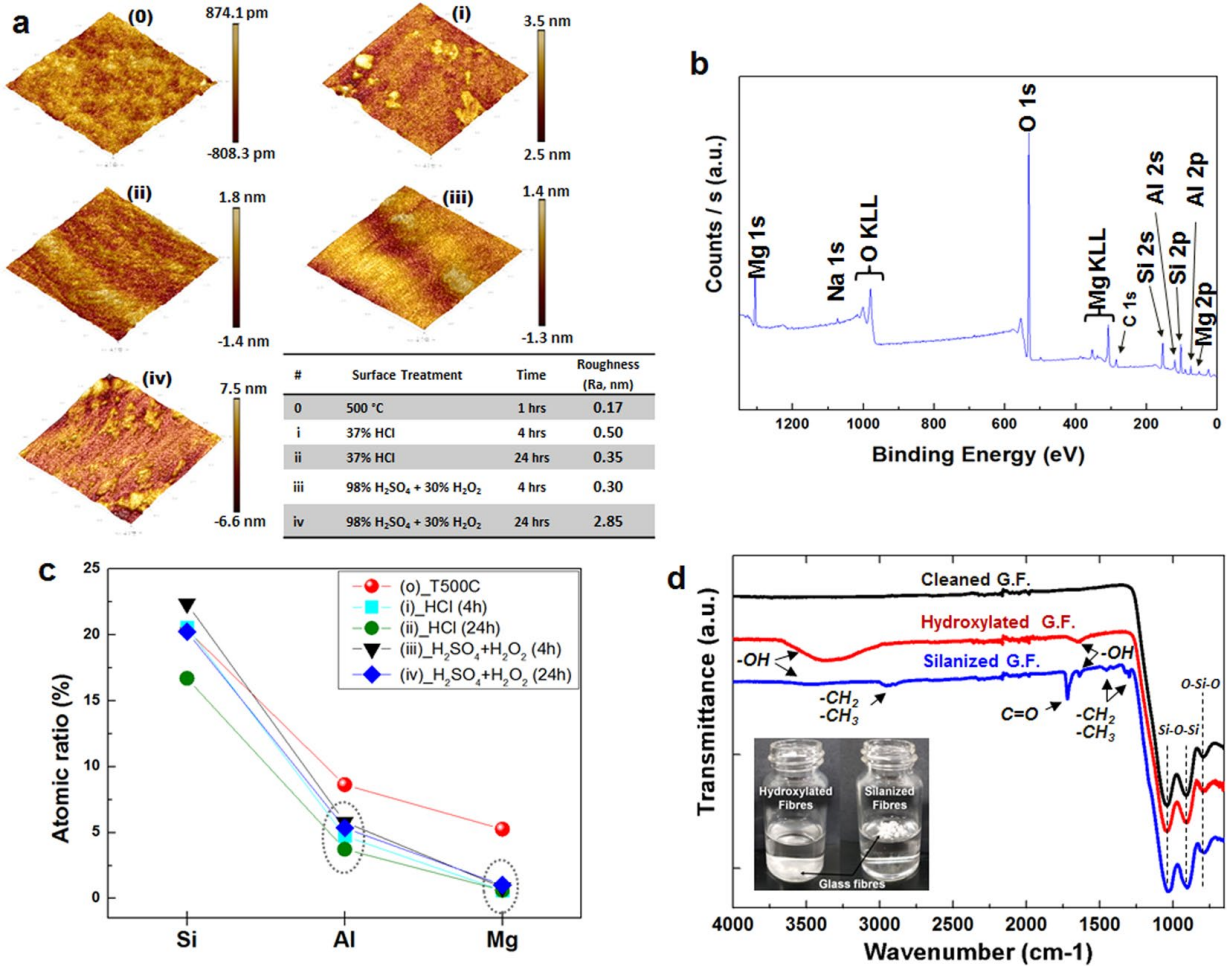
Surface characterisation of the surface modified S-Glass fibre. (**a**) AFM surface morphology of the S-Glass fibres with different etch processes which are grouped by (0)-(iv). Surface roughness (root mean square) values are described in the table. Scan area of 500 × 500 nm. (**b**) Survey spectra XPS of S-Glass fibres and (**c**) a relative atomic percentage of Si/Al/Mg in each group of glass fibres. (**d**) FTIR spectroscopy analysis of the surface modified S-Glass fibres. The inset depicts the hydroxylated glass fibres at bottom of water (left) and the silanized glass fibres floating on water (right).

The only metal components, Al and Mg, near the surface of S-Glass fibre can be selectively etched using acid solution (Fig. 1c,d) without attacking Si and SiO_2_ base material by maintaining their primitive properties^28^, and it is shown to have a negligible etching rate on Si/SiO_2_ of S-Glass fibres in micro-/nano-scale measurements (see Supplementary Fig. 3). We prepared two types of strong acid solution, 37% HCl, and a mixture of 98% H_2_SO_4_ and 30% hydrogen peroxide (H_2_O_2_) in a ratio of 3:1 which is known as ‘piranha solution’ because of its extreme corrosivity. To remove the size coating on the glass fibres without any surface damage, Group-(0) was treated in a vacuum oven for 1 hour at 500 °C, discussed elaborately in subsequent section (Method). The samples were grouped and treated in the following manner:

Group-(u) Untreated fibres;

Group-(0) Heat treatment at 500 °C for 1 hour;

Group-(i) Etched in the 37% HCl for 4 hours;

Group-(ii) Etched in the 37% HCl for 24 hours;

Group-(iii) Etched in the piranha solution for 4 hours;

Group-(iv) Etched in the piranha solution for 24 hours;

An atomic force microscope (AFM) is then used to characterise the surface tomography (Fig. 2a). To compare the surface roughness changes, Group-(0) was prepared and tested with high temperature treatment to fully remove the size coating and organic contamination on the glass fibres. The average surface roughness values (R_a_) of all groups (0-iv) were 0.17, 0.50, 0.35, 0.30 and 2.85 nm, respectively. The Group-(iv) shows almost 16 times higher value in roughness than Group-(0). A strong oxidant, H_2_SO_5_ ^29^, produced from the mixture (H_2_SO_4_ + H_2_O_2_) may dissolve the aluminium and magnesium oxides as well as some weak silicon oxide bonding for a long period time. The X-ray photoelectron spectroscopy (XPS) confirmed that S-Glass fibre consists of the elements of silicon (Si), aluminium (Al), magnesium (Mg) and oxide (O) (Fig. 2b) and has established notable differences in atomic ratio of metal elements near the glass fibre surface before and after etching treatments (Fig. 2c). The obvious decrease of Al and Mg atomic ratio in Group-(i-iv) compared to the control Group-(0) confirmed the successful reduction of metal elements during the etching process. In the control sample Group-(0), Al and Mg atomic ratio of 8.79 and 4.64 was measured and those are reduced to 3.73–5.80 and 0.60–1.02, respectively, after etching treatments. The atomic ratio of Si, Al and Mg elements was determined from Si 2p, Al 2p and Mg 1 s.

The highly etched surface can bring the potential risk of mechanical strength-degradation to the glass fibres and induce negative reinforcing effect on the resulting composites. In contrast, the increased micro/nano mechanical interlocking sites on the S-Glass fibre surface which was built by selective etching of metal oxides enhance the interfacial shear strength between fibre and matrix. Additionally, acid solution processing promotes hydroxylation on the roughened surface and on the newly exposed reactive atoms, which allow forming substantially thin and uniform coating of SCA. These are two primary approaches to effectively reinforce the short S-Glass fibre composites, and their mechanical performances are verified using direct and indirect methods, discussed in the foregoing sections of this paper.

### Molecular bridging layer - Silane coupling agent

After the etching process, glass fibres are functionalised by grafting the SCA, 3-(Trimethoxysilyl) propyl methacrylate (TMSPMA) (Fig. 1b, see Methods for the detailed synthesis). In order to form a single layer of SCA coating, 62 mg of each group fibres were mixed in 10 g of 2% TMSPMA ethanol/water solution. As the surface roughness was increased, they attract much more -OH groups on the surface and sequentially promote the adsorption capability towards SCAs^30,31^. The chemical components of the glass fibres after surface etching and TMSPMA grafting process as well as control samples were characterized using Fourier transform infrared spectrum (FTIR) in the attenuated total reflection (ATR) mode (Fig. 2d). The strong peaks near 1024 cm^−1^ and 900 cm^−1^ are assigned to Si-O-Si symmetric stretching and bending vibrations. The peak at 780 cm^−1^ is assigned to O-Si-O or Si-OH vibrations. A spectrum of surface etched glass fibres shows the wide peak in 3500–3000 cm^−1^ and small peak at 1650 cm^−1^ which identifies the presence of isolated -OH groups on the fibre surface. After TMSPMA grafting process, hydroxyl groups were consumed to form the covalent bond between fibre surface and TMSPMA, leading to a decrease in intensity peaks, and functional groups of TMSPMA are detected at 2980 cm^−1^ and 2920 cm^−1^ which are assigned to the stretching vibrations of –CH_3_ and –CH_2_ in SCA^32^.

SCA will have a strong covalent bond with the fibre surface making it hydrophobic and organophilic^33,34^. As shown in the inset of Fig. 2d, the surface-silanized glass fibres float on distilled water surface due to their hydrophobicity, but the surface-hydroxylated glass fibres by acid etching treatments attract water molecules and sink instantly into the bottom of water when they are dropped on water. The hydrophobic surface modification contributes to improving the fibre dispersion in the resin matrix (see the Supplementary Note 1). A functional group at the opposite site of silane couplant will have a covalent bond with the mono-/polymer matrix. The unique property of SCAs which possess an organic and inorganic functionality creates a strong molecular bridging layer between the glass fibre and matrix.

### Mechanical characterisation of single S-Glass fibres and reinforced composites

**Figure 3:**
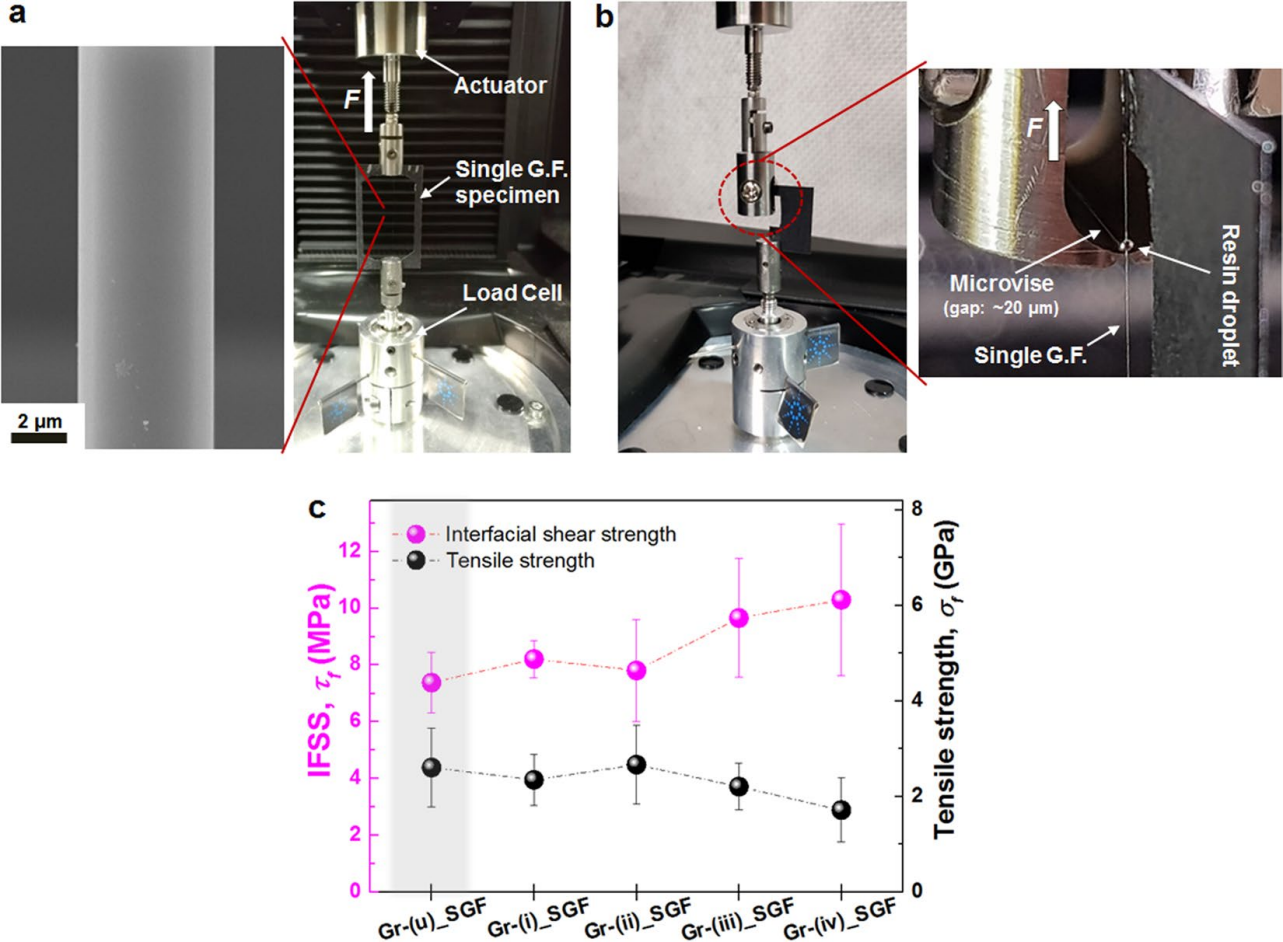
Mechanical properties of single glass fibres. **(a)** Experimental setup for the single glass fibre tensile testing and SEM image of the single glass fibre mounted on a load frame. (**b**) Experimental setup for the single fibre/microdroplet pull-out tests. (**c**) Single fibre tensile and pull-out test results. IFSS (*τ*_*i*_) in the Group-(iii) and -(iv) shows a significantly increased value up to 30.9% and 39.6%, respectively, but the tensile strength (*σ*_*f*_) in the Group-(iv) decreases showing a significant difference due to the rigorous surface etching.

For fibre reinforced composite systems, the mechanical strength of the composite has a linear relationship with strength and IFSS of the fibre (see Supplementary Note 2). As the surface of S-Glass fibres is etched and roughened to nanoscale depth using acid solutions, the mechanical strength must be testified for any degradation in properties due to the changed surface morphology. Four groups of surface treated glass fibres with etching and one control group of untreated glass fibre were prepared and their mechanical properties were carefully investigated using the single glass fibre tensile tests (Fig. 3a, see Methods, Supplementary Fig. 4 and Supplementary Movie). Statistically, the mean values of tensile strength in Group-(i), (ii) and (iii) has no significant difference from that of control group (untreated glass fibres, Group-(u)), 2.61 ± 0.83 GPa, but only the value of Group-(iv), 1.72 ± 0.67 MPa, is significantly different from all other groups showing the reduction of 34.1% in tensile strength. (Fig. 3c, see Supplementary Fig. 5). Group-(iv) showed about 6–9 times higher value in surface roughness value as shown in Fig. 2a. The reduction of strength can be attributed to the deeply etched nano-structural surface on which acute notches readily trigger the crack initiation and propagation during the tensile testing (see Supplementary Fig. 7c).

To evaluate the effect of glass fibre surface modification on interfacial properties, the changes in IFSS of fibre and resin were investigated using microdroplet pull-out tests. Microdroplet specimens were prepared by depositing a droplet of resin onto the single fibre using a micro manipulator. After light-curing, embedded length of the droplet was measured and ranged from 190 to 430 *µ*m. The droplet was positioned inside micro vise which gap is maintained with 20 *µ*m as shown in Fig. 3b, and pull-out force was measured (see Methods for detailed test setup). IFSS was calculated using the equation, ${\tau }_{i}=F/(\pi {d}_{f}{l}_{e})$, where *τ*_*i*_ refers to IFSS, *F* is the maximum load prior to the interface debonding, *d*_*f*_ is the fibre diameter and *l*_*e*_ is the fibre embedded length. The change of IFSS to the different surface treatments is shown in Fig. 3c. Group-(iii) and -(iv) show a 30.9% and 39.6% increase in IFSS compared to untreated fibres. A critical fibre length *l*_c_, which performs optimal load transfer in the composite system, can be determined by relation, ${l}_{c}={\sigma }_{f}{d}_{f}/2{\tau }_{i}$, where *σ*_*f*_ is the strength of the fibre, *τ*_*i*_ is IFSS and *d*_*f*_ is the given fibre diameter^35^ (see Supplementary Note 2). The value of critical fibre length is reduced from 916.0 *µ*m of the untreated fibres to 432.5 *µ*m of Group-(iv) fibres.

3 point-bend tests were performed on each group of composites (see Supplementary Note 3). 5 wt.% of glass fibres in each group were uniformly mixed with the mixture of 40 wt.% of strontium aluminoborosilicate glass, triethylene glycol dimethacrylate (TEGDMA) and urethane dimethacrylate (UDMA) monomers that are attractively used in dental restorative applications^17,36,37^. We successfully achieved uniform dispersion of randomly oriented short fibre composites using high-speed mixer (see Methods for the detailed fabrication). The population of fibres was calculated in the SEM cross-section images of the composites, and the obtained averaged number of fibres was 532 ± 91 fibre/mm^2^.

**Figure 4:**
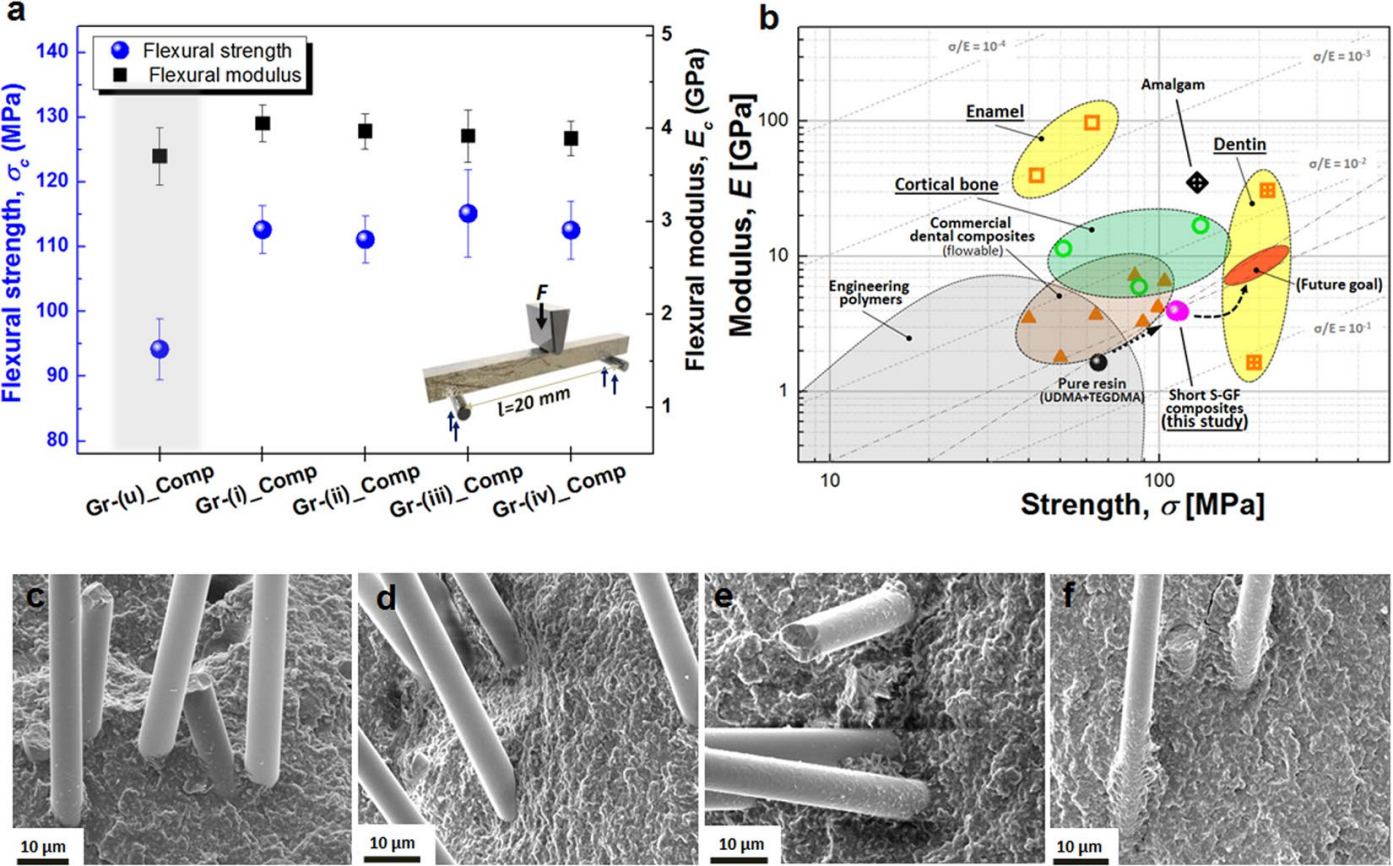
Mechanical properties of the short S-Glass fibre reinforced composites. (**a**) The flexural strength (*σ*_*c*_) and modulus (*E*) of the composites. Flexural strength of Group-(iii)_Comp which were reinforced by Group-(iii) glass fibres shows the highest value (115.15 ± 6.72 MPa) suggesting the most effective reinforcement process. (**b**) Ashby plot showing the strength-modulus values of polymeric dental composites, cortical bone, and tooth with current works. (**c**) SEM image illustrating the fracture surface of Group (i) glass fibres, (**d**) SEM image illustrating the fracture surface of Group (ii) glass fibres (**e**) SEM image illustrating the fracture surface of Group (iii) glass fibres and (**f**) SEM image illustrating the fracture surface Group (iv) glass fibres.

Interestingly, flexural strength and flexural modulus of the composites reinforced by the Group-(i)~(iv) fibres show the increase up to 22.3% and 9.7%, respectively, compared to those of the untreated fibre reinforced composite (Fig. 4a). Especially, the composite reinforced by the Group-(iii) glass fibres shows the highest value of 115.15 ± 6.72 MPa in flexural strength. Figure 4b represents the typical values of strength and modulus of polymer based dental materials^17,38,39^, human teeth (enamel and dentin)^40–44^ and cortical bones^45,46^, and current work. It can be seen that the current work achieved reasonable increase in flexural strength and modulus when compared to the commercial dental composites. There is further potential to reach near that of dentin or cortical bone. Group-(iii) glass fibres most effectively reinforce the composites via silanization because the hydroxyl (-OH) radicals generated from the mixture of H_2_SO_4_ and H_2_O_2_ facilitate the strong and uniform deposition of SCA on the glass fibres. Table 1 summarizes the strength and modulus of single glass fibres and fibre-reinforced composites. As shown in Fig. 4c–f and Supplementary Fig. 6, the glass fibre surface of the Group-(iii) and (iv) in a fractured composite is covered with more polymer residue in comparison to Group-(i) and (ii), which is a characteristic of efficient interfacial bonding and successful load transfer process in the fibre-matrix system. The flexural strength of the composites reinforced by Group-(iv) slightly decreases to 112.57 ± 4.48 MPa, which is attributed to the degraded tensile strength of glass fibre as verified at the single glass fibre tensile tests in Fig. 3c. Consequently, many fibres in the Group-(iv) composites were broken along the fracture surface of the composite instead of pulling-out (see Supplementary Fig. 7a,b). Therefore, the bond between the fibre and matrix governs whether the fibre will improve the properties of the composites while sustaining the tensile strength property of fibre in the same level as demonstrated in the combination of Group-(iii) fibres and matrix.

**Table 1:** Mechanical properties of single glass fibres and fibre-reinforced composites.

Surface treatment	Single glass fibre			Fibre-reinforced composite	
	Tensile Strength (GPa)	Elastic Modulus (GPa)	IFSS (MPa)	Flexural Strength (MPa)	Flexural Modulus (GPa)
Gr-(u)_ Untreated fibre	2.61 ± 0.83	86.15 ± 5.38	7.38 ± 1.07	94.19 ± 4.73	3.70 ± 0.31
Gr-(i)_ HCl 4 hr	2.35 ± 0.53	92.74 ± 3.70	8.21 ± 0.66	112.64 ± 3.71	4.06 ± 0.20
Gr-(ii)_ HCl 24 hr	2.67 ± 0.83	88.72 ± 7.94	7.81 ± 1.80	111.13 ± 3.58	3.97 ± 0.19
Gr-(iii)_ Piranha 4 hr	2.21 ± 0.49	88.93 ± 5.53	9.66 ± 2.09	115.15 ± 6.72	3.92 ± 0.28
Gr-(iv)_ Piranha 24 hr	1.72 ± 0.67	91.45 ± 7.62	10.30 ± 2.68	112.57 ± 4.48	3.90 ± 0.19

## Discussion and Conclusions

**Figure 5:**
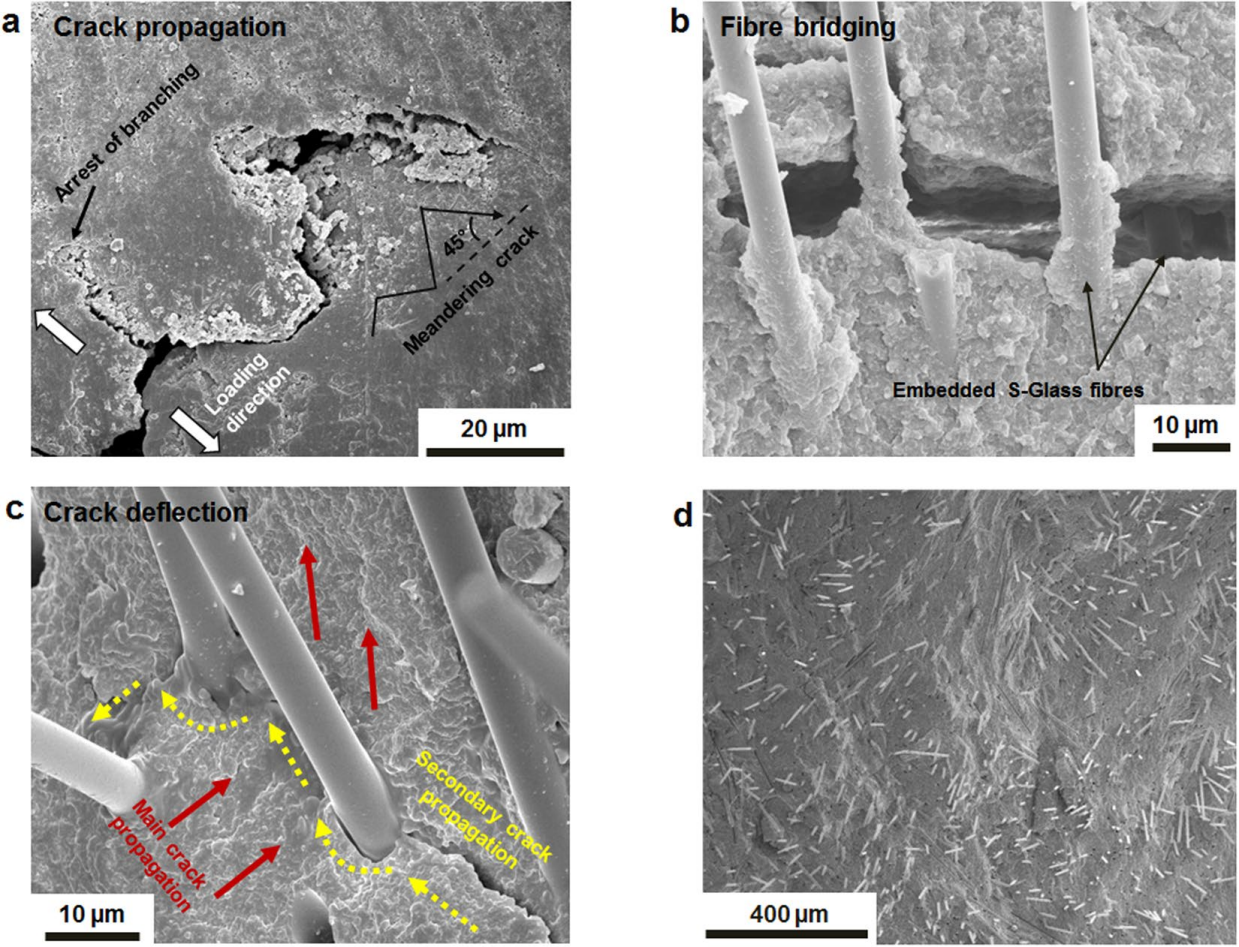
SEM micrographs of fractured surface of the composites. **(a**) Crack propagation trajectory with approximated kink angle of 45°. (**b,c**) SEM images showing the S-Glass fibre toughening mechanism for fibre bridging (**b**) and for crack deflection (**c**). (**d)** Fractured surface representing the uniformly dispersed glass fibres.

To assess the microstructural effects of embedded S-Glass fibres in composites, the crack paths were analysed near the crack-tip. The crack exhibited random fluctuation in crack profiles with kink angle of 45° (Fig. 5a). Two major reinforcement mechanisms, fibre bridging and crack deflection, are clearly investigated on the fractured surface of the newly developed composites in this study (Fig. 5b,c). Embedded short fibres generally encourage holding a crack shut as bridging crack wave and also result in a change in the crack propagation direction; both serve as potent extrinsic strengthening and toughening mechanisms^47,48^ in a fibre reinforced composite. Additionally, the hydrophobic coating of silane couplant and high-speed mixing of fibres with dental resin (see Methods) may support to scatter the fibres and to yield an almost isotropic composite structure (Fig. 5d). Uniform dispersion of short fibres in the composites will boost reinforcing efficiency. These findings may suggest that the micro scaled short glass fibres enable to develop an advanced energy consumption composite material, in which fibres will absorb mechanical energy before rupture.

In summary, with the advanced surface functionality of short S-Glass fibres we presented markedly improved mechanical properties of the polymer composites. The intensified surface functionalisation of short S-Glass fibre based on selective metal-oxide etching process preserved the tensile property of the fibre and improved the interfacial bonding in the matrix. In this method, as shown in Group-(iii), the mixture of H_2_SO_4_ and H_2_O_2_ most effectively acts as a metal oxide etchant for 4 hours as well as supplying sufficient hydroxyl group (-OH) radicals on the S-Glass fibres. The concentration of hydroxyl groups on the roughly textured glass fibre surface facilitates forming the thin layer of covalently bonded organosilicon monomer (TMSPMA) coating. It works as a strong intermediate bridge layer by effectively transferring the stress energy from matrix to fibres. Furthermore, composites are reinforced via mechanical interlocking of surface-roughened S-Glass fibres. Meanwhile, the tensile strength and IFSS of single glass fibres which are treated with strong acid solutions are measured to investigate on the optimal condition of the etching process. We demonstrated that dental resins reinforced by surface functionalised short S-Glass fibres exhibit an increase in the flexural strength (22.3%) and flexural modulus (9.7%) compared to those with the resins reinforced by untreated S-Glass fibres. In technical and commercial respects, the surface modification process we developed is simple in its processing and selective metal etching on S-Glass fibres may open up new perspectives to use the commercial glass fibres in various composite applications. While this study used dental resins to develop new flowable dental composites, our method will lead to the development of new biomaterials and composites with advanced mechanical and physical properties.

## Methods

### Materials

A commercially available S-Glass fibre, marketed as S-2 type glass fibres (AGY, USA) were used for this investigation. Glass fibres (5 µm diameter) chopped to a length of 250 µm are procured from Engineered Fibers Technology, USA. The length and diameter were measured by SEM, and one-way analysis of variance (ANOVA, Minitab7) was used to confirm that the mean length and diameter of the glass fibres were the same with our target mean value 250 *μ*m and 5 *μ*m, respectively (see Supplementary Fig. 1). Energy-dispersive spectrometry (EDS) analysis was carried out to verify the material composition in weight % of S-2 glass fibre which contained a mixture of SiO_2_ (65%), Al_2_O_3_ (25%) and MgO (10%). A dental resin obtained from SDI Limited, Australia, is prepared by mixing 80 wt.% of urethane dimethacrylate (UDMA), 20 wt.% of triethylene glycol dimethacrylate (TEGDMA) and 0.2 wt.% of camphoroquinone (CQ), 0.5 wt.% of ethyl 4-dimethylaminobenzoate (EDMAB) and 0.05 wt.% of butylated hydroxyl-toluene (BHT) inhibitor. Additionally, strontium aluminoborosilicate glass with a particle size of 0.7 *μ*m were used as fillers in the dental resins obtained from SDI.

### Surface functionalisation of S-Glass fibres

Two acid-etching solutions, 37% hydrochloric acid (HCl) and 98% sulphuric acid (H_2_SO_4_) aqueous solution are used to etch the glass fibre surface. To increase the density of hydroxyl groups on the glass fibre surface, 30% hydrogen peroxide (H_2_O_2_) was mixed with H_2_SO_4_ in a ratio of 1:3. When preparing the mixture of H_2_SO_4_ with H_2_O_2_, the hydrogen peroxide was added to sulphuric acid slowly with stirring. This reaction is very exothermic and can raise the temperature above 100 °C, which requires cooling in an ice bath when mixed. Glass fibres were etched in each of the two acid solutions at room temperature for 4 hours and 24 hours with stirring at 250 rpm. Most acid solution was removed and fibres are neutralized with saturated sodium bicarbonate aqueous solution. Neutralized fibres were rinsed with deionized water and acetone followed by drying in an oven at 90 °C for 24 hours. 62 mg of completely dried fibres in each group was treated in 10.0 g of SCA solution, which consists of 2% 3-(trimethoxysilyl) propyl methacrylate silane (TMSPMA, Sigma-Aldrich, MO, USA) in 93% ethanol, 5% deionized water and 2~3 drops of acetic acid to titrate to pH of 3~4, for 1 hour with stirring at 500 rpm at room temperature. The fibres, then, were rinsed with deionized water and ethanol. Fibres in aqueous solutions in each process were filtered out using a centrifugation separation at 6,000 rpm for 3 minutes and subsequently dried at 90 °C for 24 hours. The control samples were prepared by removing the size coating and organic contamination on the glass fibres in a high temperature vacuum oven at 500 °C for 1 hour.

### Characterisation

Surface micrographs of all resulting fibres and fracture surface of composites were examined under the FE-SEM (Nova NanoSEM 450 FE-SEM). To quantify the morphology and topography of the etched glass fibres, AFM imaging in tapping mode (AFM, Bruker Multimode 8) was used. Surface roughness and images were acquired in 500 × 500 nm area with a line scan rate of 0.5 Hz. All samples were measured in tapping mode and the ScanAyst-Air AFM cantilever (the nominal tip radius of 2 nm, the nominal cantilever resonant frequency of 70 kHz and the nominal spring constant of 0.4 N/m) was used. The organic functional groups on the glass fibres which were grafted with SCA were identified using a Perkin-Elmer (Wiesbaden, Germany) FTIR spectrometer in universal attenuated total reflection mode (ATR). FTIR scans were carried out in the range from 650 to 4000 cm^−1^ at 4 cm^−1^ resolution. The X-ray photoelectron spectroscopy (Thermo ESCALAB250Xi) was used to examine changes of surface elements of S-Glass fibres.

### Single glass fibre tensile and pull-out test

Tensile and pull-out tests on single glass fibres were performed using the Agilent T159 UTM which has 500 mN load range and 5 nN load resolution. Specimens for single fibre tensile test were prepared according to ISO-11566 standard using a custom approach described in Supplementary Fig. 4a. All tests were performed with a cross head speed of 0.45 mm/min through the linear actuator until the final fracture of the fibre occurs. For the microdroplet pull-out test, a micro vise was manufactured using a wire electrical discharge machining (WEDM) to create a fine contact surface, and a gap width of 20 *µ*m was maintained during the tests. Resin droplets were light-cured for 1 minute using the same light source as preparing the composites. Pull-out test was also conducted with a cross head speed of 0.45 mm/min.

### Synthesis of glass fibre reinforced composites and flexural tests

The prepared dental resin (mixture of UDMA/TEGDMA/CQ/EDMAB/BHT) was successively mixed with 40 wt.% of strontium aluminoborosilicate glass and 5 wt.% of surface functionalised S-Glass fibres using a high-speed mixer (Speedmixer DAC 150.1 FVZ-K, FlackTek Inc., Landrum, USA) at 3500 rpm for 20 seconds three times. Mixed resin must be held in vacuum pressure of 95.0 kPa for 1 minute to remove the micro-bubbles which were created during the turbulent mixing (see the Supplementary Fig. 8). Thereafter, the mixed resin was shaped in the metal moulds (2.0 × 2.0 × 25.0 mm: width, height and length), and was cured by blue light (Radii Plus, SDI Limited, Australia) with wavelength ranges of 440 to 480 nm and intensity of 1,500 mW/cm^2^. The three-point bend tests were conducted using a universal testing machine (Instron 3369, Instron Ltd., USA) on the light cured dental composites for the flexural tensile strength and flexural modulus.

## Supplementary information

Supplementary Movie

Selective Atomic-Level Etching on Short S-Glass Fibres to Control Interfacial Properties for Restorative Dental Composites
