# Supplementary material for: Selective Atomic-Level Etching on Short S-Glass Fibres to Control Interfacial Properties for Restorative Dental Composites
Source: Sci Rep. 2019 Mar 7;9:3851. doi: 10.1038/s41598-019-40524-7 (PMC6405923; doi:10.1038/s41598-019-40524-7)
Supplement: Supplementary file 2 — Selective Atomic-Level Etching on Short S-Glass Fibres to Control Interfacial Properties for Restorative Dental Composites [file 41598_2019_40524_MOESM2_ESM.docx]

**Supplementary Information**

**Selective Atomic-Level Etching on Short S-Glass Fibres to Control Interfacial Properties for Restorative Dental Composites**

Kiho Cho^1*^, Guannan Wang^2^, Raju^1^, Jian Fang^3^, Ginu Rajan^4^, Martina H. Stenzel^2^, Paul Farrar^5^ & B. Gangadhara Prusty^1*^

**



**

**b**

**d**

**c**

**a**

**



**

**e**

**f**


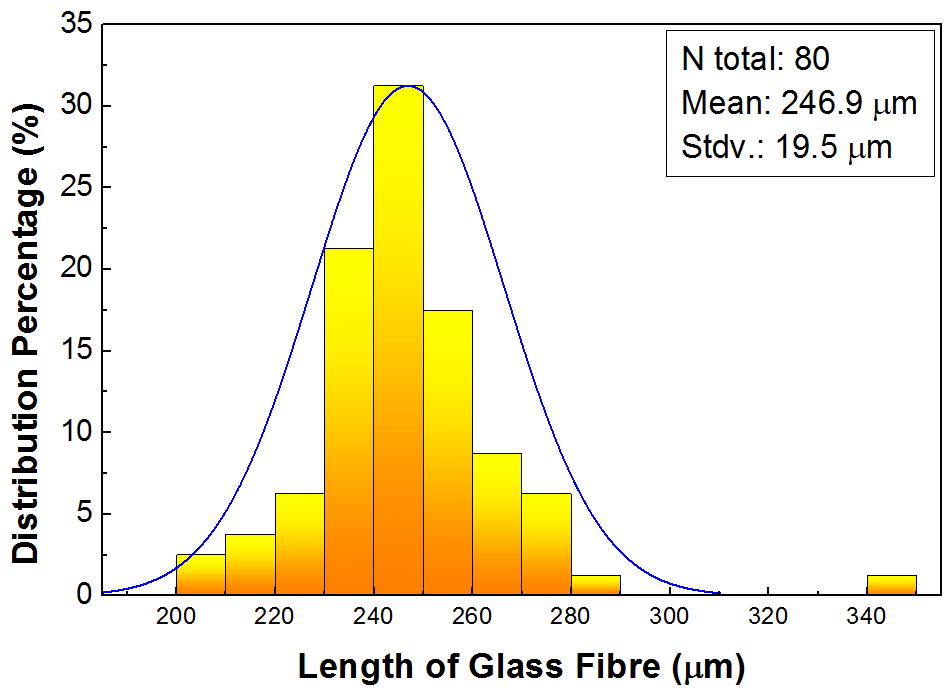

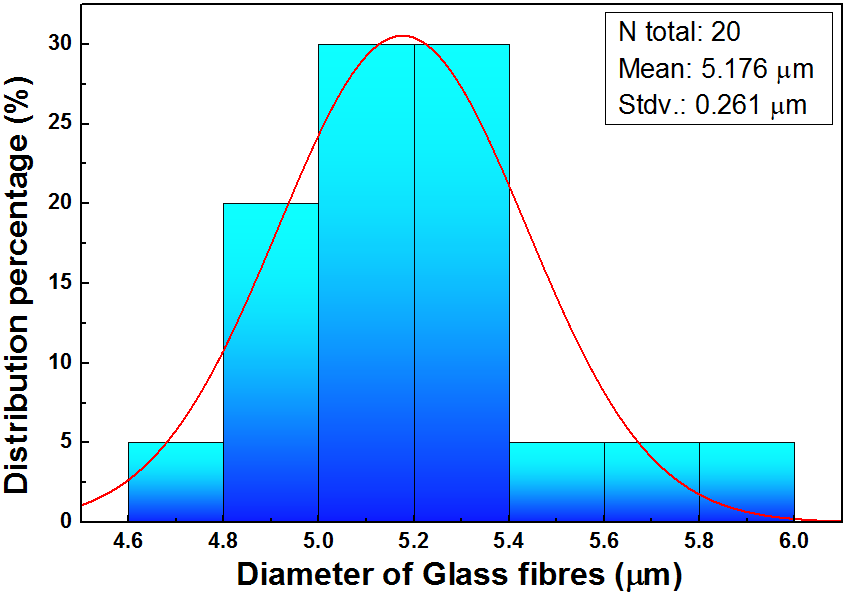


**Supplementary Figure 1. Statistical characterization of short S-2 glass fibres. (a-b)** SEM images of S-2 glass fibres which are coated by sizing. **(c-d)** SEM images of S-Glass fibres which are treated with 37% HCl solution for 30 minutes to remove the sizing layer only. The sizing layer and glass debris are clearly removed that allows measuring the length and diameter of the fibres. **(e)** The length and **(f)** diameter distribution histograms of short S-Glass fibres generated by measuring approximately 80 and 20 glass fibres in representative SEM images.


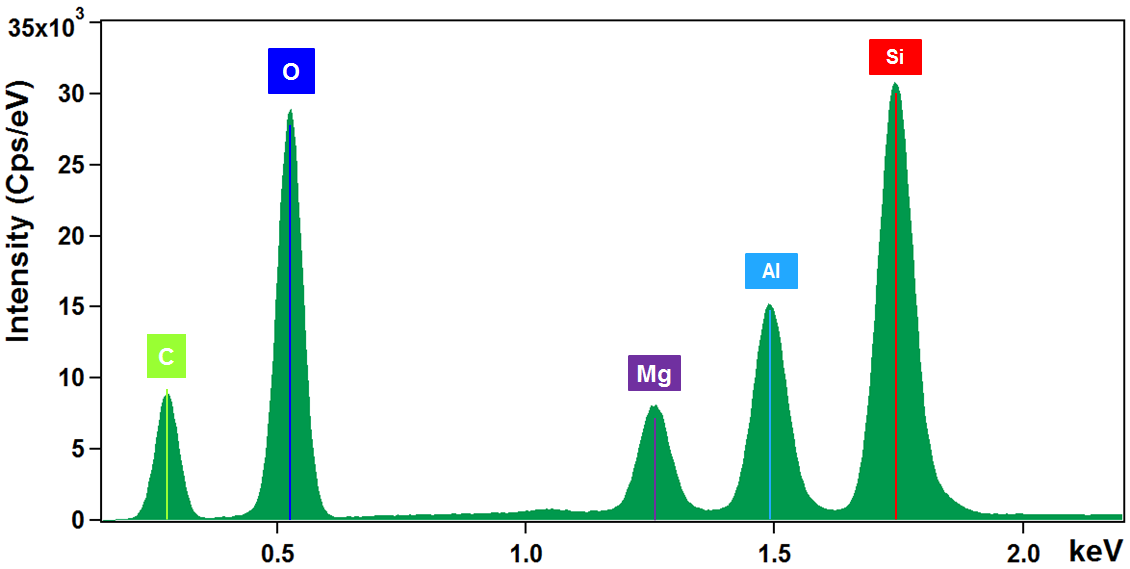


**a**

**c**

**b**


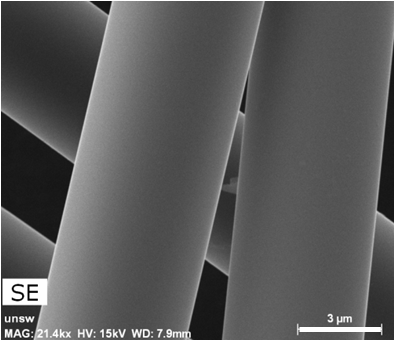

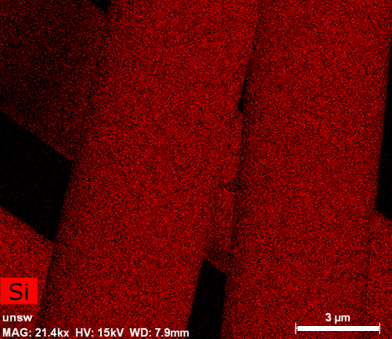


**e**


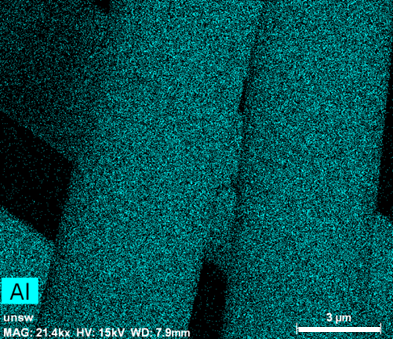

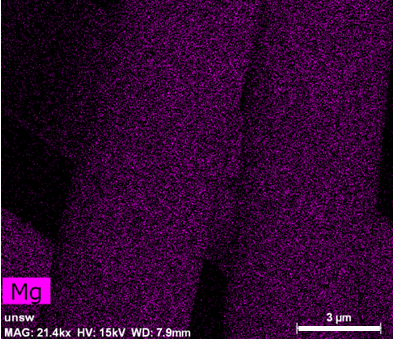


**f**

**d**


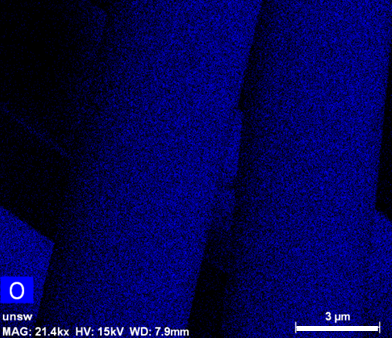


**Supplementary Figure 2. The material composition analysis of S-Glass fibres. (a)** Energy dispersive spectrometer (EDS) spectrum of S-Glass fibres in which the elements of Si, Al, Mg and O are distinctly detected. Carbon element in the spectrum comes from the pre-treated carbon sputter coating (Emitech, K575x) which makes the glass fibres surface electrically conductive. **(b-f)** SEM–secondary electron (SE) image **(a)** and SEM energy-dispersive spectrometry (EDS) element maps of Si **(c)**, Al **(d)**, Mg **(e)** and O **(f)**. Scale bars in **b-f**: 3 μm.


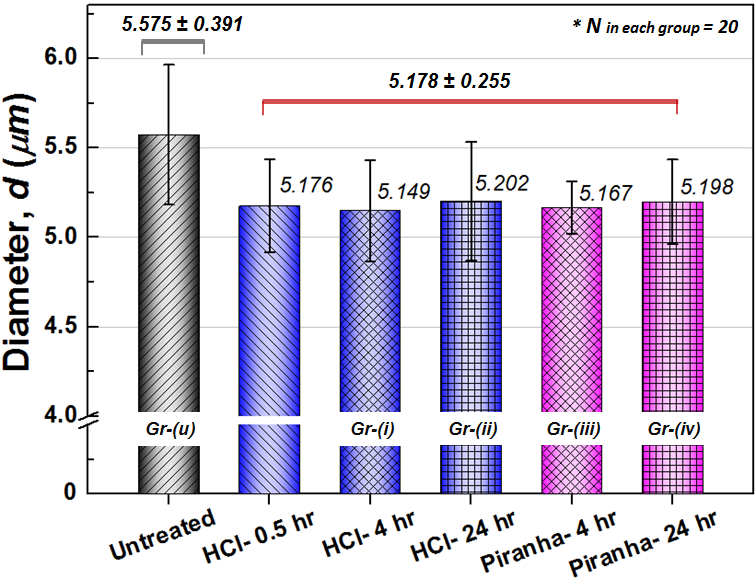


**Supplementary Figure 3. Diameter measurement of S-Glass fibres.** Mean value of the glass fibre diameters before and after etching process was measured using SEM images and statistically analysed using one-way ANOVA. A polymeric size coating (~0.2 µm in thickness) on untreated fibres (black column) was easily removed by acid etching solutions, and the diameter of glass fibre significantly reduced from 5.575 ± 0.391 µm to 5.178 ± 0.255 µm (p < 0.05). Any significant change in glass fibre diameter with continued etching process was not detected even after 24 hours etching process (blue and pink columns) (p > 0.05). The result strongly supports that a chemical structure of SiO_2_ in S-Glass fibres has strong resist against HCl and piranha acid solutions and only metal components at the near surface of S-Glass fibres were etched in atomic- and nano-scale which was also verified using AFM and XPS measurements (see Fig. 2a-c).

**b**

**a**


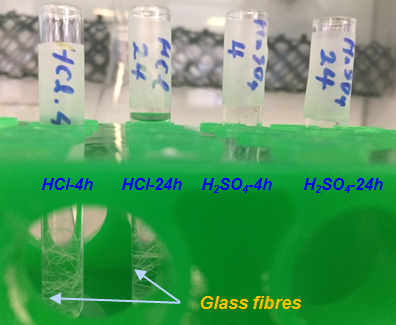

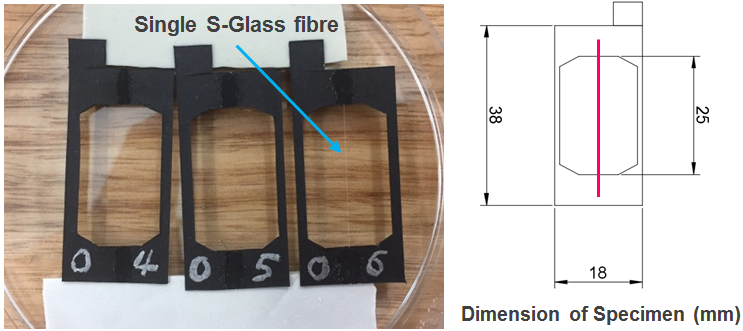


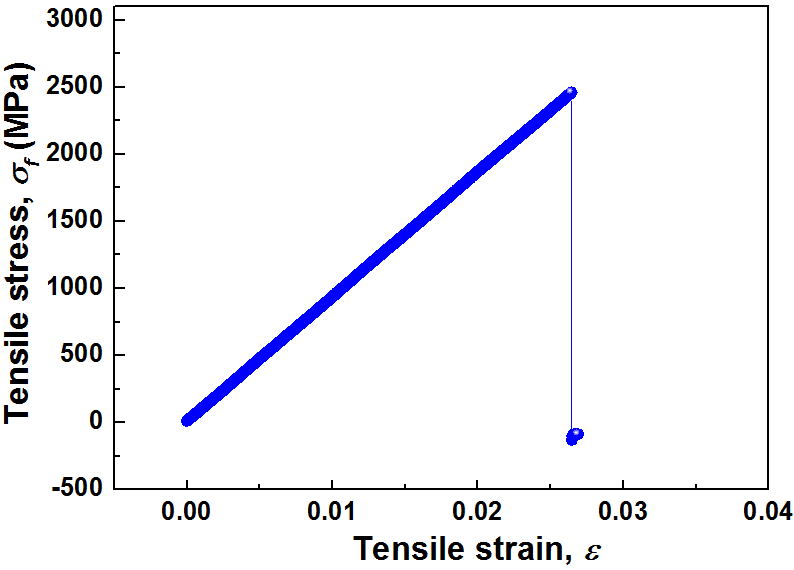


**c**

**Supplementary Figure 4. Sample preparation for the single glass fibre tensile tests. (a)** Long glass fibres are soaked in acid solutions, 37% HCl and the mixture of 98% H_2_SO_4_ and 30% H_2_O_2_ for 4 hours and 24 hours. **(b)** Preparation of specimens for the tensile test following international standard ISO-11566. After rinsing, a separated single glass fibre is fixed on the paper template with a gauge length of 25 mm. **(c)** Tensile stress vs. strain curves of the unprocessed single S-Glass fibre.


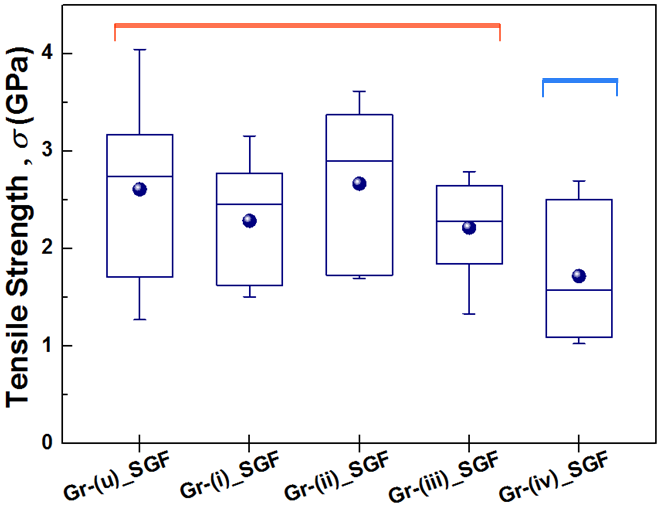


**Supplementary Figure 5. Statistical analysis of single glass fibre (SGF) tensile test results.** Groups within horizontal lines (colored in red) are not significantly different at the significance level of 0.05. Only Group-(iv) shows a significant difference in the mean value of tensile strength from other groups which attributes to the roughly etched surface with the mixture of H_2_SO_4_ and H_2_O_2_ solution for 24 hours. Supplementary Table 1-4 details the results of statistical analysis.

**b**

**a**


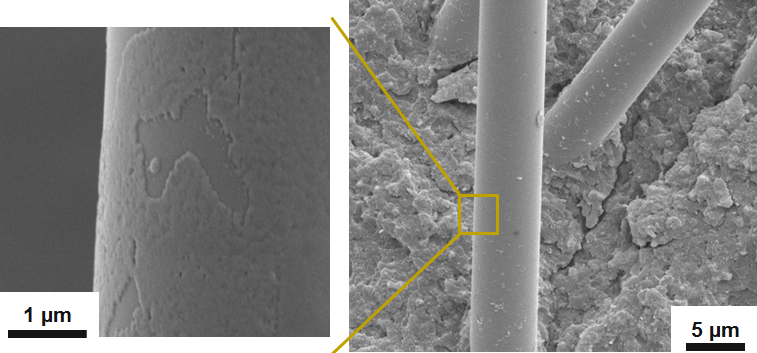

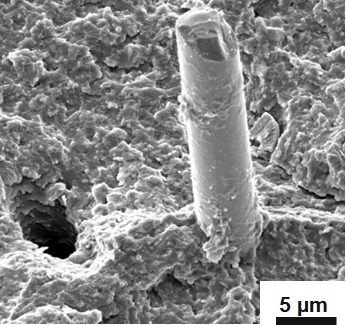


**Supplementary Figure 6.** SEM images of S-Glass fibre surface etched with HCl for 4 hours (**a**) and H_2_O_2_/H_2_SO_4_ mixture (Piranha solution) for 4 hours (**b**).


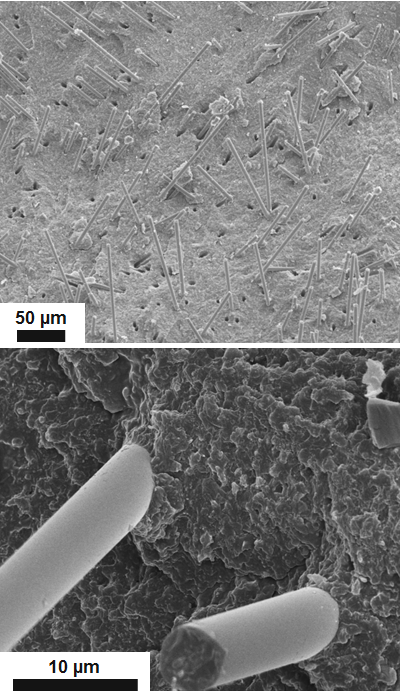

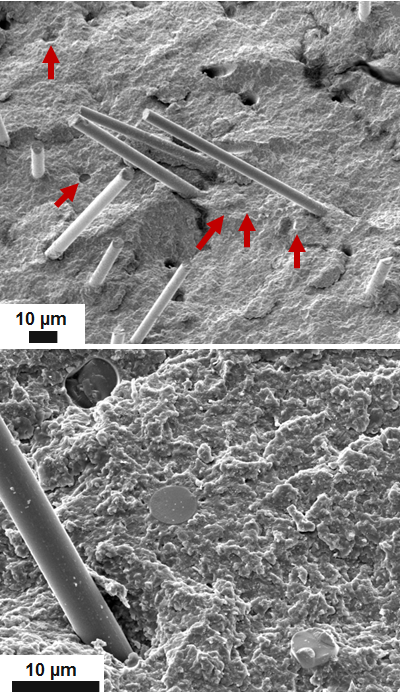


**b**

**a**


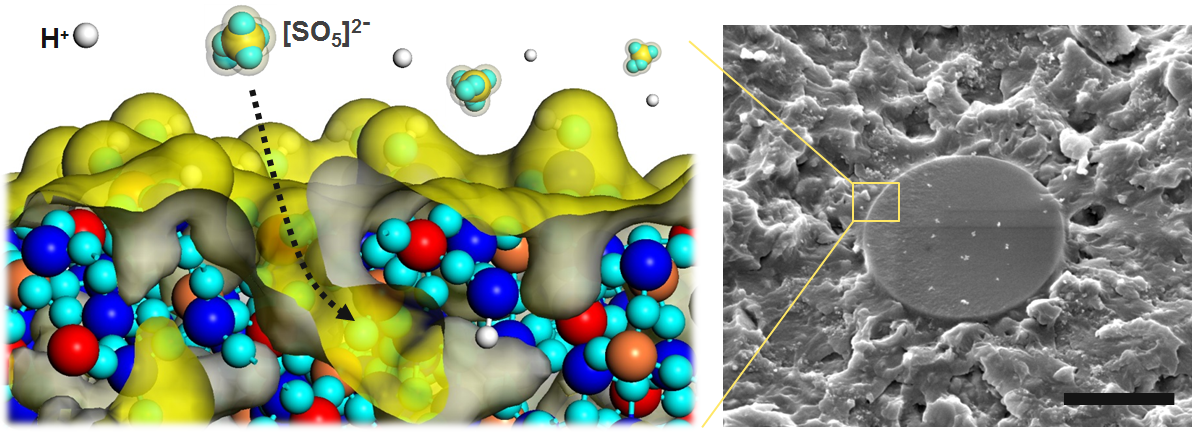


**c**

**Supplementary Figure 7. SEM images of the fracture surface. (a)** The fractured surfaces of the composites reinforced with Group-(i) which is etched in 37% HCl for 4 hours and **(b)** with Group-(iv) which is rigorously etched in the mixture of 98% H_2_SO_4_ and 30% H_2_O_2_ solution for 24 hours. **(c)** Atomic model of rigorous deep etching on the near surface of S-Glass fibre. In figure **(b)**, many fibres do not pull out but break instead which may explain the reduced tensile strength (*σ_c_*) of composites fibres due to the degraded tensile strength (*σ_f_*) of fibres as described in Supplementary Note 3. Scale bars in **c**: 3 µm.

**b**

**a**


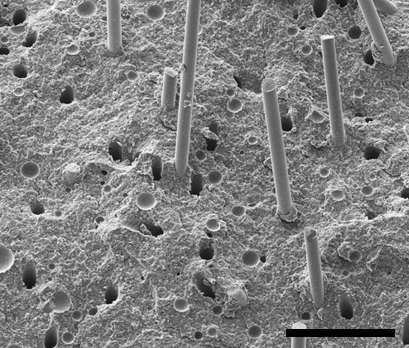

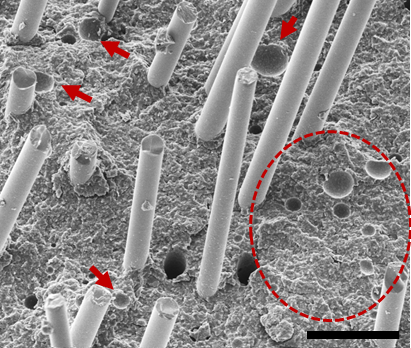


**d**

**c**


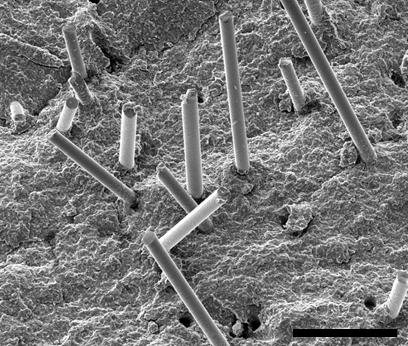

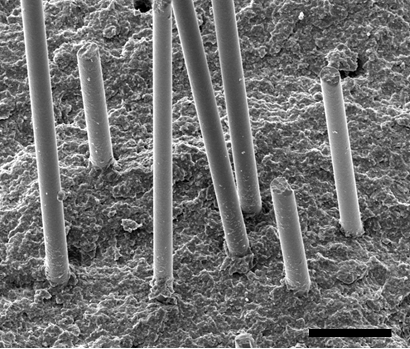


**Supplementary Figure 8. Cross sectional SEM images of the composites before and after vacuum infusion process. (a, b)** Micro voids in the matrix caused by entrained air during the high speed mixing. (**c, d**) Voids are removed using moderate vacuum infusion. Scale bars in **a,b**: 40 µm and **c,d**: 20 µm.

**Supplementary Table 1. Tensile strength results of single S-Glass fibres (SGF)**

| **Group** | **N Analysis** | **Mean (MPa)** | **Standard Deviation (MPa)** |
| --- | --- | --- | --- |
| **Group-(u)_SGF** | 11 | 2607.9 | 834.3 |
| **Group-(i)_SGF** | 12 | 2356.0 | 530.1 |
| **Group-(ii)_SGF** | 8 | 2665.9 | 832.2 |
| **Group-(iii)_SGF** | 8 | 2213.3 | 486.8 |
| **Group-(iv)_SGF** | 10 | 1717.8 | 674.4 |

**Supplementary Table 2. Results of one-way ANOVA with all groups of single glass fibre tensile tests:** P-value (0.035) is less than 0.05. Therefore, at the 0.05 level, we reject the null hypothesis that the means of all levels are equal and conclude that the population means are significantly different.

|  | **DF** | **Sum of Squares** | **Mean Square** | **F Value** | **P-Value** |
| --- | --- | --- | --- | --- | --- |
| **Model** | 4 | 5.56E+06 | 1.39E+06 | 2.834 | **0.035** |
| **Error** | 44 | 2.16E+07 | 490543.1084 |  |  |
| **Total** | 48 | 2.71E+07 |  |  |  |

**Supplementary Table 3. Results of one-way ANOVA with the untreated glass fibres (Gr-(u)) and Group (i)-(iii) glass fibres:** P-value (0.429) is higher than 0.05. Therefore, at the 0.05 level, we cannot reject the null hypothesis and conclude that the population means are not significantly different.

|  | **DF** | **Sum of Squares** | **Mean Square** | **F Value** | **P-Value** |
| --- | --- | --- | --- | --- | --- |
| **Model** | 3 | 1.42E+06 | 472442.7 | 0.945 | **0.429** |
| **Error** | 35 | 1.75E+07 | 499722.4 |  |  |
| **Total** | 38 | 1.89E+07 |  |  |  |

**Supplementary Table 4. Results of one-way ANOVA with the Group-(u) and Group-(iv) glass fibres:** P-value (0.015) is less than 0.05. Therefore, at the 0.05 level, we reject the null hypothesis and conclude that the population means are significantly different.

|  | **DF** | **Sum of Squares** | **Mean Square** | **F Value** | **P-Value** |
| --- | --- | --- | --- | --- | --- |
| **Model** | 1 | 4.15E+06 | 4.15E+06 | 7.131 | **0.015** |
| **Error** | 19 | 1.11E+07 | 581828.914 |  |  |
| **Total** | 20 | 1.52E+07 |  |  |  |

**Supplementary Note 1: Grafting silane coupling agent on the S-Gglass fibre**

The organofunctional silane coupling agents have been widely used in surface functionalisation techniques improving the interfacial bonding of fibres and nanotubes^40,41^. They work as a chemical bridge to couple the inorganic materials to organic mono-/polymers. Therefore, we have selected 3-(Trimethoxysilyl)propyl methacrylate (TMSPMA), H_2_C=C(CH_3_)CO_2_(CH_2_)_3_Si(OCH_3_)_3_, as a silane couplant in this study. Hydrolysis chemical reactions of TMSPMA in a water-ethanol solution are described below[^42^](#_ENREF_3) .

H_2_C=C(CH_3_)CO_2_(CH_2_)_3_Si(OCH_3_)_3_ → H_2_C=C(CH_3_)CO_2_(CH_2_)_3_Si(OH)_3_ + 3CH_3_OH **(S1)**

*n*H_2_C=C(CH_3_)CO_2_(CH_2_)_3_Si(OH)_3_ → HO[H_2_C=C(CH_3_)CO_2_(CH_2_)_3_Si(OH)_2_ ]_n_ + *n*H_2_O **(S2)**

TMSPMAs first react with water and produce highly reactive silanol groups. Subsequently, they start forming a self-condensed film on the fibres while also forming weak hydrogen bonds to the hydroxyl (-OH) groups on the glass fibre surface which was chemically etched and enriched by hydroxyl groups. Finally, during drying at 90 °C, the hydrogen bonds between the two sets of hydroxyl groups are transformed to the Si-O-Si covalent bonds causing the dihydroxylation^43,44^.

**Supplementary Note 2: Mechanical relationship between short fibre and composites**

In the fibre reinforced composite systems, the strength (*σ_c_*) and toughness (*G_c_*) of composites are strongly related with the mechanical properties of the embedded fibres such as volume fraction (*f_f_*), length (*l*) and diameter(*d*), tensile strength (*σ_f_*), and interfacial shear strength (*τ_i_*) of fibres. A critical fibre length (*l_c_*) can be approximately calculated by relation (S5)^46^. A critical fibre length is defined as the shortest effective fibre length transferring the external load from matrix to fibre in a composite system and the value of *l_c_* depends on parameters such as tensile strength of fibre and interfacial strength between fibre and matrix. In this study, the surface properties of short S-Glass fibres are tailored by selective etching and silane coupling agent coating preserving the tensile strength of fibres but increasing the interfacial shear strength, so the functionalised fibres can increase the strength and toughness of composites based on the equation S6 and S7 (as we neglect the atomic scale change of the diameter during the etching and coating process).

$l_{c}= \frac{\sigma_{f} d}{2\tau_{i}}$ **(S5)**

$\sigma_{c}= f_{f}\sigma_{f} \left( 1-\frac{l_{c}}{2l} \right)+ f_{m}\sigma_{m}$ **(S6)**

$G_{c}= \pi d \tau_{i} \frac{l^{2}}{8} \times\frac{4f_{f}}{\pi d^{2}}= \frac{f_{f}}{2d} \tau_{i} l^{2}$ **(S7)**

Where *f_f_* and *f_m_* refer to the volume fraction of fibre and matrix respectively in the composite.

**Supplementary Note 3: Flexural strength and modulus of composites**

To evaluate the flexural strength of composites, 3-point bend tests were conducted employing a universal testing machine (Instron 3369, Instron Ltd., USA) with a displacement rate of 1 mm/min. The loading and pushing pin both had diameter of 2mm and test span was set at 20 mm. Flexural strength (*σ*) was determined using the following equation^45^:

$\sigma= \frac{3P_{m}L}{2Wt^{2}}$ **(S3)**

where, *P_m_* is the maximum load at crack extension, *L* is the length between spans, *W* is the specimen width, and *t* is the specimen thickness.

The flexural modulus (*E*) was computed using the following equation^45^:

$E= \frac{SL^{3}}{4wt^{3}}$ **(S4)**

where, *S (ΔF/Δd)* is the initial slope of the load-displacement curve, *F* is the load and *d* is the deflection.

**Supplementary References**

40 Xie, Y. J., Hill, C. A. S., Xiao, Z. F., Militz, H. & Mai, C. Silane coupling agents used for natural fiber/polymer composites: A review. *Compos Part a-Appl S* **41**, 806-819 (2010).

41 Ma, P. C., Kim, J. K. & Tang, B. Z. Functionalization of carbon nanotubes using a silane coupling agent. *Carbon* **44**, 3232-3238 (2006).

42 Wei, B. G. *et al.* Surface modification of filter medium particles with silane coupling agent KH550. *Colloid Surface A* **434**, 276-280 (2013).

43 Abdelmouleh, M., Boufi, S., Belgacem, M. N. & Dufresne, A. Short natural-fibre reinforced polyethylene and natural rubber composites: Effect of silane coupling agents and fibres loading. *Compos Sci Technol* **67**, 1627-1639 (2007).

44 Yuk, H., Zhang, T., Lin, S. T., Parada, G. A. & Zhao, X. H. Tough bonding of hydrogels to diverse non-porous surfaces. *Nat Mater* **15**, 190 (2016).

45 Low, I. M., McGrath, M., Lawrence, D., Schmidt, P., Lane, J., Latella, B. A., & Sim, K. S. Mechanical and fracture properties of cellulose-fibre-reinforced epoxy laminates. *Composites Part A: Applied Science and Manufacturing*, **38**(3), 963-974 (2007).

46 Bagherpour, S. Fibre reinforced polyester composites. *Polyester*. (Intech, 2012).
